# The Cytochrome P450 Epoxygenase Pathway Regulates the Hepatic Inflammatory Response in Fatty Liver Disease

**DOI:** 10.1371/journal.pone.0110162

**Published:** 2014-10-13

**Authors:** Robert N. Schuck, Weibin Zha, Matthew L. Edin, Artiom Gruzdev, Kimberly C. Vendrov, Tricia M. Miller, Zhenghong Xu, Fred B. Lih, Laura M. DeGraff, Kenneth B. Tomer, H. Michael Jones, Liza Makowski, Leaf Huang, Samuel M. Poloyac, Darryl C. Zeldin, Craig R. Lee

**Affiliations:** 1 Division of Pharmacotherapy and Experimental Therapeutics, Eshelman School of Pharmacy, University of North Carolina, Chapel Hill, North Carolina, United States of America; 2 Division of Intramural Research, National Institute of Environmental Health Sciences, National Institutes of Health, Research Triangle Park, North Carolina, United States of America; 3 Department of Pharmaceutical Sciences, School of Pharmacy, University of Pittsburgh, Pittsburgh, Pennsylvania, United States of America; 4 Division of Molecular Pharmaceutics, Eshelman School of Pharmacy, University of North Carolina, Chapel Hill, North Carolina, United States of America; 5 Department of Pathology and Laboratory Medicine, School of Medicine, University of North Carolina, Chapel Hill, North Carolina, United States of America; 6 Department of Nutrition, Gillings School of Global Public Health, University of North Carolina, Chapel Hill, North Carolina, United States of America; INRA, France

## Abstract

Fatty liver disease is an emerging public health problem without effective therapies, and chronic hepatic inflammation is a key pathologic mediator in its progression. Cytochrome P450 (CYP) epoxygenases metabolize arachidonic acid to biologically active epoxyeicosatrienoic acids (EETs), which have potent anti-inflammatory effects. Although promoting the effects of EETs elicits anti-inflammatory and protective effects in the cardiovascular system, the contribution of CYP-derived EETs to the regulation of fatty liver disease-associated inflammation and injury is unknown. Using the atherogenic diet model of non-alcoholic fatty liver disease/non-alcoholic steatohepatitis (NAFLD/NASH), our studies demonstrated that induction of fatty liver disease significantly and preferentially suppresses hepatic CYP epoxygenase expression and activity, and both hepatic and circulating levels of EETs in mice. Furthermore, mice with targeted disruption of *Ephx2* (the gene encoding soluble epoxide hydrolase) exhibited restored hepatic and circulating EET levels and a significantly attenuated induction of hepatic inflammation and injury. Collectively, these data suggest that suppression of hepatic CYP-mediated EET biosynthesis is an important pathological consequence of fatty liver disease-associated inflammation, and that the CYP epoxygenase pathway is a central regulator of the hepatic inflammatory response in NAFLD/NASH. Future studies investigating the utility of therapeutic strategies that promote the effects of CYP-derived EETs in NAFLD/NASH are warranted.

## Introduction

Non-alcoholic fatty liver disease (NAFLD) is a rapidly growing public health problem that is prevalent in approximately 30% of the United States general population [1]. NAFLD begins with simple steatosis, and may progress to non-alcoholic steatohepatitis (NASH), and ultimately to advanced fibrosis and cirrhosis of the liver [2]. Although the progression from NAFLD to NASH is poorly understood, the development and progression of hepatic inflammation is a key pathological mediator in this transition and is associated with the development of comorbid conditions [3], [4].

In the early stages of NAFLD, an imbalance between uptake and export of lipids by hepatocytes leads to lipid accumulation within the liver. Increased hepatic saturated fatty acids and cholesterol activate toll-like receptors (TLRs) that drive activation of nuclear factor-κB (NF-κB)-mediated inflammatory responses [5]. Sustained activation of the hepatic inflammatory response drives macrophage infiltration into the liver, ultimately causing fibrosis and hepatic injury [6]. Consistent with this pathological progression of NAFLD/NASH, the high-fat/high-cholesterol “atherogenic” diet model of steatohepatitis induces dyslipidemia, hepatic inflammation, and fibrosis, through an innate immune-mediated mechanism [7], [8].

Arachidonic acid is metabolized by cyclooxygenases, lipoxygenases, and cytochromes P450 (CYP) to biologically active eicosanoids, which are critical regulators of numerous biological processes including inflammation [9]. CYP enzymes are abundantly expressed in the liver where they catalyze the oxidative biotransformation of xenobiotics [10]. In addition, certain CYP isoforms metabolize endogenous substrates. Notably, CYP epoxygenase enzymes from the CYP2C and CYP2J subfamilies metabolize arachidonic acid to biologically active epoxyeicosatrienoic acids (EETs) [11]. However, EETs are rapidly hydrolyzed by soluble epoxide hydrolase (sEH, *Ephx2*) to their corresponding dihydroxyeicosatrienoic acid (DHETs), which are generally less biologically active [11]. Previous studies have shown that acute, lipopolysaccharide (LPS)-evoked activation of the innate immune response suppresses hepatic CYP epoxygenase expression and EET biosynthesis [12]. Moreover, increased endothelial EET biosynthesis, or decreased global sEH-mediated EET hydrolysis, attenuates NF-κB activation and the acute vascular and systemic inflammatory response to LPS [12], [13], [14].

Collectively, these studies demonstrate that hepatic EET biosynthesis is suppressed in response to activation of the innate immune system, and potentiation of the CYP epoxygenase pathway attenuates innate immune-dependent acute inflammatory responses [15]. However, the functional relevance of the CYP epoxygenase pathway in the development and progression of sustained, fatty liver disease-associated hepatic inflammation and injury has not been investigated. Therefore, the objective of our study was to 1) evaluate the impact of atherogenic diet-induced NAFLD/NASH on hepatic CYP epoxygenase expression and EET biosynthesis; and, 2) determine if promoting the effects of CYP epoxygenase-derived EETs attenuates fatty liver disease-associated hepatic inflammation and injury.

## Materials and Methods

### Ethics Statement

All studies were completed in accordance with the *Public Health Service Policy on Humane Care and Use of Laboratory Animals*, and were approved by the Institutional Animal Care and Use Committee at the University of North Carolina-Chapel Hill (UNC) and the National Institute of Environmental Health Sciences.

### Reagents

Reagents were obtained from ThermoFisher Scientific (Waltham, MA) unless otherwise indicated.

### Animals

All experiments were performed in adult male mice on a C57BL/6J background that were 11–20 weeks of age at the initiation of the experiments. Wild-type (WT) C57BL/6J mice were purchased from Jackson Laboratory (Bar Harbor, ME). Mice with targeted disruption of *Ephx2* (*Ephx2^−/−^*) were backcrossed onto a C57BL/6J genetic background for more than 10 generations, as described [16]. All mice had free access to food and water and were housed in temperature and humidity controlled rooms using a 12 hour light/dark cycle.

### Experimental Protocol

Mice were fed *ad libitum* a commercially available atherogenic (ATH) diet [17], [18] containing 40% kilocalories from fat, 1.25% cholesterol and 0.5% cholic acid (D12109c, Research Diets Inc., New Brunswick, NJ) or a standard chow (STD) diet containing 14% kilocalories from fat and 0.02% cholesterol (ProLab RMH 3000, PMI Nutrition International, Brentwood, MO). An initial time-course experiment was conducted to assess the relative induction of hepatic inflammation and injury following two, four, or eight weeks of atherogenic diet administration. All subsequent studies were conducted over four weeks.

The first series of experiments evaluated the effect of atherogenic diet feeding on hepatic CYP epoxygenase expression and EET biosynthesis in WT mice (Jackson Laboratory). The second series of experiments evaluated the effect of disruption of sEH-mediated EET hydrolysis on atherogenic diet induced hepatic inflammation and injury in *Ephx2^−/−^* and corresponding WT control mice. At the termination of each experiment, mice were euthanized by CO_2_ asphyxiation. Blood was collected via cardiac puncture, and plasma was separated by centrifugation. Liver tissue was harvested; one part was snap-frozen in liquid nitrogen and stored at −80°C pending analysis, while the remainder was fixed in 4% paraformaldehyde and embedded in paraffin for histological analysis.

### Hydrodynamic Delivery of *Ephx2* to *Ephx2* ^−/−^ Mice

In order to characterize the contribution of hepatic sEH to circulating EET levels *in vivo*, the hydrodynamic injection-based transfection method was utilized to restore hepatic *Ephx2* expression in *Ephx2^−/−^* mice. In this well-characterized method, a large volume of plasmid DNA is rapidly injected into the tail vein to markedly increase hydrostatic pressure in the inferior vena cava and preferentially drive transgene delivery to the liver [19], [20].

The murine *Ephx2* (NM_007940) cDNA was PCR amplified with specific forward (5′-ATGGCGCTGCGTGTAGCC-3′) and reverse (3′-CTAAATCTTGGAGGTCAC-5′) primers from whole liver total cDNA, subcloned into the pcDNA3.1(–) expression vector (Invitrogen, Carlsbad, CA, USA) downstream of the cytomegalovirus (CMV) enhancer-promoter, and then confirmed by sequencing. *Ephx2^−/−^* mice received 0.75 mg/kg of plasmid DNA (pcDNA3.1-*Ephx2* or empty pcDNA3.1 vector [control], n = 4 per group) in phosphate buffered saline (PBS) by tail vein injection. The injection volume was 9% of the body weight (22.5 µg plasmid delivered in 2.7 mL PBS per 30 gram mouse), and the injection time was 5 seconds, as described [19], [20], [21]. After 18 hours, the mice were euthanized by CO_2_ asphyxiation, and plasma and liver tissue were harvested and stored at −80°C pending analysis.

### Direct Quantification of CYP-Derived Eicosanoids in Plasma and Liver

Arachidonic acid metabolites from the CYP epoxygenase pathway (8,9-EET, 11,12-EET, 14,15-EET, 5,6-DHET, 8,9-DHET, 11,12-DHET, 14,15-DHET) and CYP ω-hydroxylase pathway (20-hydroxyeicosatetraenoic acid [HETE]) were extracted from plasma and liver and then quantified by liquid chromatography-tandem mass spectrometry (LC-MS/MS) as previously described, with minor modifications [22]. Briefly, plasma (200 uL) was diluted in 250 uL of a 0.1% acetic acid/5% methanol solution containing 0.009 mM butylated hydroxytoluene (BHT), spiked with internal standards (3 ng each of PGE2-d4, 10,11-DiHN, and 10,11-EpHep; Cayman Chemical, Ann Arbor, MI), and then underwent solid-phase extraction, as described [22]. Following serial passage through HyperSep Retain SPE columns (Thermo Scientific, Bellefonte, PA, USA), the columns were washed and then eluted with 0.5 mL of methanol and 1 mL of ethyl acetate into glass tubes with 10 µL of trapping solution (30% glycerol in methanol). Liver tissue (approximately 20 mg) was weighed and homogenized in 0.1% acetic acid/5% methanol solution containing 0.009 mM BHT and 100 µM (5% w/v) of the sEH inhibitor *trans*-4-[4-(3-adamantan-1-ylureido)-cyclohexyloxy]-benzoic acid (*t*-AUCB, kindly provided by Dr. Bruce Hammock, University of California-Davis), spiked with the internal standards, and then underwent liquid-liquid extraction. Liver lysates were vortexed with 2 mL of ethyl acetate (twice) for 10 minutes, and the organic layers were transferred to a single glass collection tube containing 6 uL of 30% glycerol. Plasma and liver extracts were dried under nitrogen gas, purged with argon gas, and then stored at -80°C pending analysis.

Following reconstitution in 50 uL of 30% ethanol, online liquid chromatography of extracted samples was performed with an Agilent 1200 Series capillary HPLC (Agilent Technologies, Santa Clara, CA). Separations were achieved using a Halo C18 column (2.7 µm, 100×2.1 mm; MAC-MOD Analytical, Chadds Ford, PA), which was held at 50°C and a flow rate of 400 µl/min. Mobile phase A was 0.1% acetic acid in 85∶15 water:acetonitrile. Mobile phase B was 0.1% acetic acid in acetonitrile. Gradient elution was used and the mobile phase was varied as follows: 20% B at 0 min, ramp from 0 to 5 min to 40% B, ramp from 5 to 7 min to 55% B, ramp from 7 to 13 min to 64% B. From 13 to 19 min the column was flushed with 100% B at a flow rate of 550 µl/min before being returned to starting conditions and equilibrated for 6 minutes. Samples were solvated in 50 µl of 30% ethanol and injected in triplicate at 10 µl per injection.

Electrospray ionization tandem mass spectrometry was used for detection. Analyses were performed on an MDS Sciex API 3000 equipped with a TurboIonSpray source (Applied Biosystems, Foster City, CA). Turbo desolvation gas was heated to 425°C at a flow rate of 6 L/min. All analytes were monitored simultaneously in a scheduled multiple reaction monitoring experiment as negative ions at parent ion-product ion mass/charge ratio pairs and retention times. The relative response ratios of each analyte were used to calculate concentrations, while correcting for surrogate losses via quantification relative to internal standards. Liver eicosanoid concentrations were normalized to tissue weight, the 14,15-EET:DHET ratio was calculated as an *in vivo* biomarker of sEH metabolic function [16], and the sum of EET levels (8, 9-, 11, 12–14, 15-EET) were calculated as a biomarker of CYP epoxygenase pathway function [23].

### Formation of CYP-Derived Eicosanoids in Liver Microsomes

Microsomal fractions were isolated from hepatic tissue as previously described [12] and microsome protein concentrations were determined using the BCA method [24]. Microsomal protein was incubated with 50 µM arachidonic acid in 1 mL of a 0.12 M potassium phosphate incubation buffer containing 5 mM MgCl [12], and incubations were completed at 37°C for 20 minutes; reactions were initiated by adding 1 mM NADPH and terminated by placing the samples on ice. Incubations were completed in the presence of 5 µM of *t*-AUCB to minimize EET hydrolysis. Following termination of the reaction samples were diluted 10-fold, internal standard (20-HETE-d6) was added and metabolites were extracted, dried under a stream of nitrogen gas and reconstituted for analysis. Metabolites of arachidonic acid were then quantified by LC-MS/MS, as described [12]. The incubations were performed under saturating concentrations of arachidonic acid, where formation rates reflect the amount of biologically active protein and significantly correlate with CYP mRNA and protein levels [12], [25].

### Hepatic Microarray and Gene Set Enrichment Analysis

Total RNA was isolated from homogenized liver (n = 4 per group) using the RNeasy mini-prep kit (Qiagen, Valencia, CA). Global gene expression analysis was conducted using the Agilent Whole Mouse Genome 4×44 multiplex array (Agilent Technologies, Inc., Santa Clara, CA) according to the manufacturer’s protocol. Briefly, 1.65 micrograms of Cy3 labeled cRNAs were fragmented and hybridized for 17 hours in a rotating hybridization oven. Slides were then washed and scanned, data were acquired using the Agilent Feature Extraction software version 9.5 (Agilent Technologies) using 1-color defaults for all parameters. The resulting data were processed and analyzed using Genespring version 12.1 (Agilent Technologies). Only probes flagged “detected” in at least 75% of samples from at least one treatment group were included, resulting in 31,643 probes in the final dataset. Datasets were deposited with the Gene Expression Omnibus of the National Center for Biotechnology Information (GEO Series accession number GSE53381).

Gene set enrichment analysis (GSEA) Version 2.0 was used to determine if the arachidonic acid metabolism pathway is enriched in WT mice administered the atherogenic compared to WT mice administered the standard diet. Genes involved in the metabolism of arachidonic acid to biologically active eicosanoids were identified using the Kyoto Encyclopedia of Genes and Genomes (KEGG) [26] ‘arachidonic acid metabolism pathway’ for mice (map00590), and limited to genes involved in arachidonic acid liberation from the cell membrane and metabolism.

Analysis of variance (ANOVA) with unequal variance was used to generate the complete list of genes significantly up-regulated or down-regulated in response to the atherogenic diet at a false discovery rate (Benjamini-Hochberg) of 1% using Genespring GX (Agilent Technologies, Inc., Santa Clara, CA) (Table S1). Pathway enrichment was determined using the National Institutes of Health (NIH) Database for Annotation, Visualization and Integrated Discovery (DAVID) Version 6.7, which uses a modified Fisher’s Exact Test to calculate an “Enrichment Score” for each functional annotation cluster and identifies enriched biological themes without a pre-specified hypothesis [27], [28]. Biological Process Terms and Molecular Function Terms were included as Gene Ontologies, and the Classification Stringency was set to high; all other default parameters were unchanged. The list of significantly up-regulated or down-regulated genes was uploaded to DAVID and compared to the *mus musculus* reference to generate a list of functional pathways that were over-represented in response to atherogenic diet feeding. P-values were calculated by computing 10^−Enrichment Score^, with p<0.01 (Enrichment Score >2) considered statistically significant.

### Biochemical Analysis

Plasma total cholesterol and alanine aminotransferase (ALT) levels were quantified using a Vitros 350 automated chemical analyzer (Ortho-Clinical Diagnostics, Rochester, NY). Total cholesterol and triglyceride levels were quantified in homogenized liver tissue using the Biovision Cholesterol Ester Quantification Kit II and the Biovision Triglyceride quantification Kit, respectively, according to the manufacturer’s instructions (Biovision Incorporated, Milpitas, CA).

### Quantitative RT-PCR

Quantitative RT-PCR was performed in triplicate using the ABI 7300 Real-Time PCR system, as described [12]. Expression of hepatic *Tlr4* (Mm00445274_m1), *Cyp2c29* (Mm00725580_s1), *Cyp2c50* (Mm00663066_gH), *Cyp2c55 (*Mm00472168_m1), *Cyp2j5* (Mm00487292_m1), *Ephx2* (Mm01313813_m1), *Tnfa* (Mm00443258_m1), *Ccl2* (Mm00441242_m1), *Nfκb1* (Mm00476361_m1) and *Col3a1* (Mm01254476_m1) was quantified using Taqman Assays on Demand (Applied Biosystems), normalized to *Gapdh* (endogenous control, Mm99999915_g1) and expressed relative to the control group using the 2^−ΔΔCt^ method [29].

### ELISA

Liver tissue was homogenized in lysis buffer (50 mM Tris-HCl (pH 7.4), 150 mM NaCl, 1 mM EDTA, 1% Triton X, 1 mM NaF, 0.25% Na deoxycholate and protease inhibitors) and the S9 fraction was separated by centrifugation. Protein concentrations of the homogenate were quantified using the BCA method [24]. Monocyte chemoattractant protein-1 (MCP-1), and vascular cellular adhesion molecule-1 (VCAM-1) protein levels were quantified in liver homogenates using the mouse CCL2/JE/MCP-1 and VCAM-1/CD106 Quantikine ELISA kits (R&D Systems, Minneapolis, MN, USA), respectively, after loading equal amounts of protein into each well. Concentrations were normalized to mg of liver protein. Plasma concentrations of MCP-1 were quantified using the mouse CCL2/JE/MCP-1 Quantikine ELISA kit (R&D Systems) following a four-fold dilution.

### Immunoblotting

Liver homogenates (30 µg protein) were separated by 10% NuPAGE Bis-Tris gels, and then transferred to nitrocellulose membranes (Invitrogen). Membranes were blocked in 5% non-fat milk in Tris-buffered saline (TBS), washed, and then incubated with either anti-phospho-IκBα (1∶500 in 1% BSA in TBS with 0.05% Tween 20; #2859, Cell Signaling Technology, Danvers, MA, USA), anti-sEH (1∶1000 in 5% milk in TBS with 0.1% Tween 20; sc22344, Santa Cruz Biotechnology, Santa Cruz, CA), or anti-GAPDH (1∶1000 in 5% BSA in TBS with 0.05% Tween 20; #2118, Cell Signaling) antibodies, washed and then incubated with the appropriate horseradish peroxidase-conjugated secondary antibody (Santa Cruz Biotechnology), as described [12]. Immunoreactive bands were detected by chemiluminescence using the ECL Western Blotting Substrate (Thermo Scientific, Rockford, IL). The density of the immunoreactive phospho-IκBα bands, normalized to GAPDH, was quantified using ImageJ software (NIH) as a biomarker of NF-κB activation, as described [12].

### Histology and Immunohistochemistry

Liver tissue (left lobe) was fixed in 4% paraformaldehyde for 24 hours, processed, embedded in paraffin, and then sectioned using a serial interrupted technique (5 µm sections, 200 µm apart). Sections underwent hematoxylin and eosin (H&E) and immunohistochemical (F4/80) staining. Digital images were acquired with the ScanScope CS slide capture device (Aperio, Vista, CA) and analyzed using ImageScope Version 11.1 (Aperio).

Immunohistochemistry was performed by treating slides with pH 6.0 sodium citrate buffer (Dako North America, Carpinteria, CA) and endogenous peroxide was quenched by placing slides in a solution of 3% H_2_O_2_ for 15 minutes. Sections were blocked using 0.25% casein in PBS (Dako North America) then incubated with a rabbit anti-F4/80 polyclonal antibody at a 1∶200 dilution (Santa Cruz Biotechnology) followed by a goat anti-rabbit secondary antibody at a 1∶500 dilution (Jackson Immunoresearch, West Grove, PA). Antibody binding was detected using Vectastain Elite ABC Kit (Vector Laboratories, Burlingame, CA) and visualization was performed with 3,3-diaminobenzidine. Sections were then counterstained with hematoxylin, dehydrated, and mounted.

Macrophage infiltration was quantified by counting the number of inflammatory foci (i.e., the number of macrophage clusters) per 10x field, as described [30]. All analyses were performed in duplicate by the same individual who was blinded to treatment group. Three non-overlapping fields were analyzed across three sections; the number of foci were then summed and averaged to obtain an estimate for each mouse.

The presence and extent of NAFLD was evaluated on hematoxylin and eosin (H&E) stained slides according to a standardized and well-established histological scoring system (NAFLD Activity Score) that evaluates the presence and severity of steatosis, lobular inflammation, hepatocyte ballooning, and fibrosis [31]. Lobular inflammation, which is the most relevant histological consequence of the atherogenic diet [8], was graded in blinded fashion by a pathologist (H.M.J.) according to the number of inflammatory infiltrates per 200x field as follows: 0 (no infiltrates); 1 (<2 infiltrates per field); 2 (2–4 infiltrates per field); and 3 (>4 infiltrates per field).

### Statistical Analysis

Data were normalized to the atherogenic diet treated WT control group and pooled across experiments, unless otherwise indicated, and expressed as mean ± standard error of the mean (SEM). For continuous variables, rank-transformed mean values were compared across diet and genotype groups using a one-way ANOVA followed by Fisher's LSD post hoc test with p<0.05 considered statistically significant. Lobular inflammation score (0, 1, 2, or 3) was compared across groups by Chi-squared test with a post-hoc ordinal logistic regression analysis. Correlation between continuous variables was evaluated using Spearman’s rank correlation. All statistical analyses were performed using SAS version 9.3 (SAS Institute, Cary, NC).

## Results

### Characterization of the Atherogenic Diet Model of NAFLD/NASH

We first conducted a time-course experiment to characterize the effects of atherogenic diet administration over time. Atherogenic diet administration for two, four or eight weeks significantly increased hepatic *Ccl2* mRNA levels (MCP-1, a chemokine that recruits monocytes; 3.0±0.9-, 10.9±4.1-, and 7.9±2.7-fold, respectively) and plasma ALT levels (a biomarker of hepatocellular injury; 3.6±0.5-, 4.0±0.5- and 2.9±0.3-fold, respectively) compared to the standard chow diet (p<0.005 for each comparison). Since hepatic inflammation and injury were not further increased at eight weeks, the four-week time-point was utilized for all follow-up studies.

**Figure 1:**
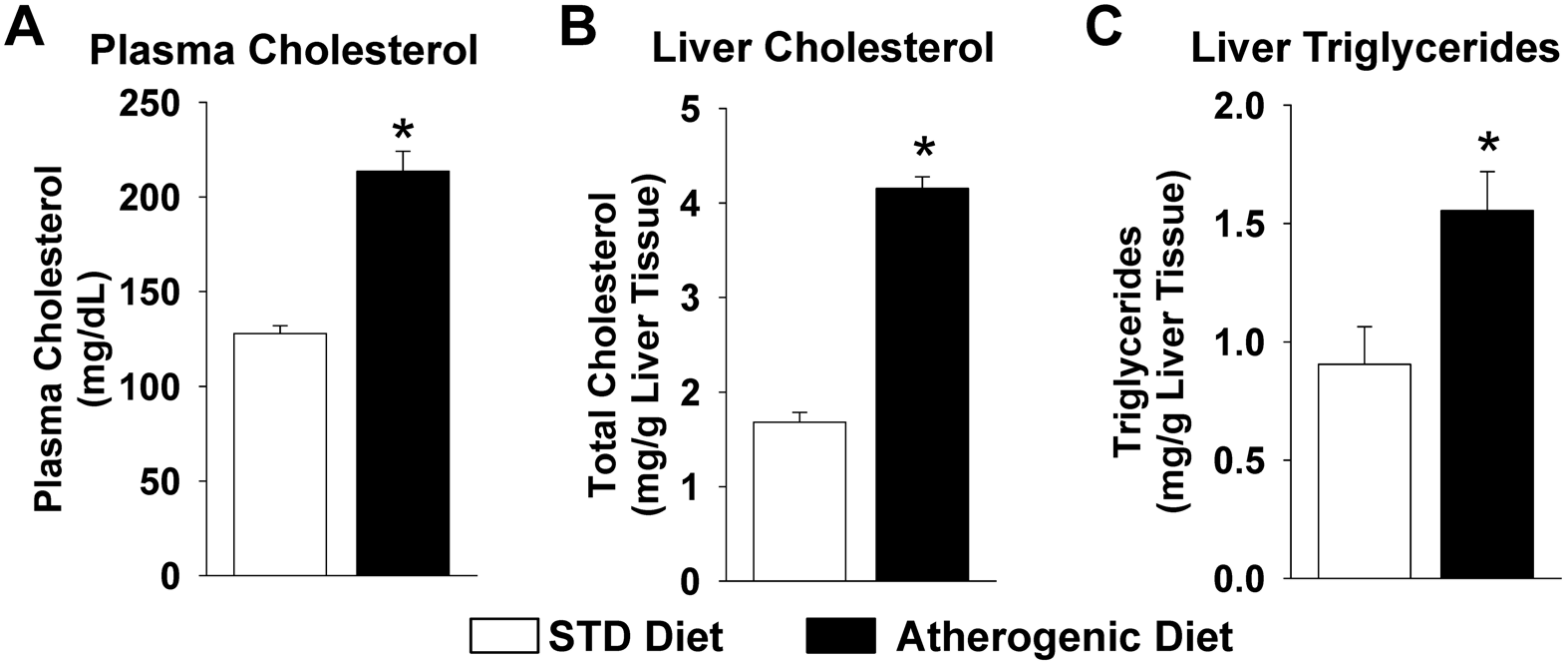
Plasma and hepatic lipid levels increase in response to atherogenic diet administration. (A) Plasma total cholesterol levels, (B) liver total cholesterol levels, and (C) liver triglyceride levels were significantly higher in mice administered the atherogenic diet for 4 weeks compared to mice administered the STD diet (STD diet: n = 6–10, atherogenic diet: n = 13–22). *P<0.05 vs. STD diet group.

At four weeks, plasma total cholesterol, hepatic total cholesterol, and hepatic triglyceride levels were significantly higher in mice administered the atherogenic diet compared to mice administered the standard chow diet (Figure 1). Body weight did not change significantly from baseline (−0.2±0.2 grams, p = 0.397) in response to the atherogenic diet; however, mice administered the standard diet experienced modest weight gain (1.2±0.2 grams, p<0.001). Collectively, these data demonstrate that the atherogenic diet model induces hepatic lipid accumulation, inflammation, and injury, which are key drivers of the development and progression of NAFLD/NASH, independent of weight gain.

### Differential Hepatic Expression of Arachidonic Acid Metabolism Pathway Genes

**Figure 2:**
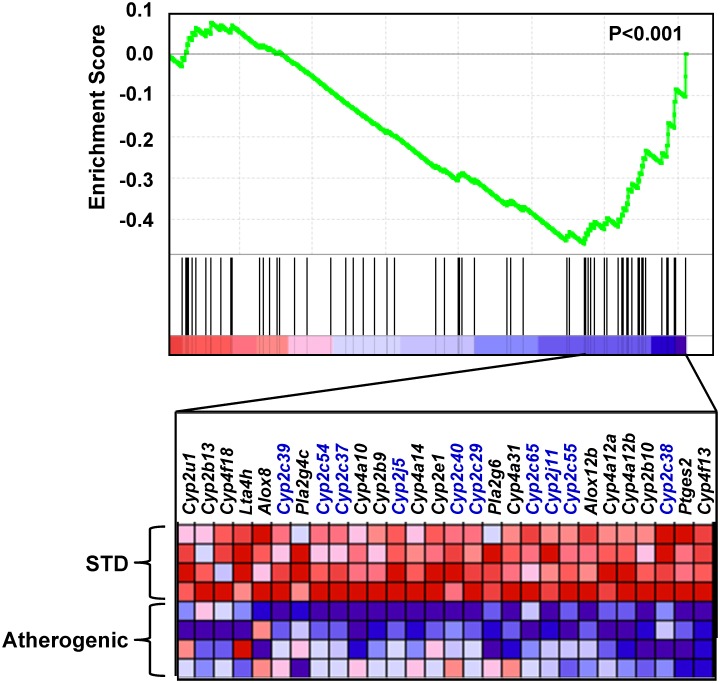
Hepatic expression of arachidonic acid metabolism pathway genes in response to atherogenic diet administration. Gene expression profiling was completed in liver using the Agilent Whole Mouse Genome Microarray (n = 4 per group). To generate the enrichment plot, each gene on the microarray is rank-ordered (left to right) according to its correlation with atherogenic diet administration (most positive on the far left, most negative on the far right). The enrichment plot for the arachidonic acid metabolism pathway gene set enrichment analysis (GSEA) indicates the position of each gene (vertical lines) within the pathway in the overall rank-order of the correlation (top of figure). The p-value for the GSEA is provided. A heat-map is provided illustrating gene expression levels for each gene in the core enrichment subset (bottom of figure). Blue indicates the gene is down-regulated and red indicates the gene is up-regulated. CYP epoxygenase genes from the Cyp2c and Cyp2j subfamilies are denoted in blue.

We subsequently sought to determine whether the arachidonic acid metabolism pathway was differentially expressed in liver tissue in WT mice administered the atherogenic diet. Of the 82 genes in the arachidonic acid metabolism pathway, 67 (82%) were included on the microarray and passed our quality control standards. GSEA demonstrated that the arachidonic acid metabolism pathway was significantly enriched in mice administered the atherogenic diet (p<0.001). Moreover, the core enrichment subset, which is the subset of genes accounting for the observed signal, was down-regulated in mice administered the atherogenic diet compared to mice administered the standard chow diet (Figure 2), indicating that relative to global hepatic gene expression changes, the arachidonic acid metabolism pathway was suppressed in liver following atherogenic diet administration. Notably, 22 of the 28 (79%) genes included in the core enrichment subset were CYP transcripts, and 10 of the 22 CYPs (46%) were CYP epoxygenases from the Cyp2c and Cyp2j subfamilies. In parallel, biological pathway enrichment was quantified using the full list of genes that were significantly up-regulated or down-regulated in liver by atherogenic diet feeding in order to identify enriched biological themes without a pre-specified hypothesis. Consistent with the GSEA results, the “cytochrome P450” cluster was among the most significantly enriched pathways (Table S2).

### Dysregulation of Cytochrome P450-Mediated Eicosanoid Metabolism

**Figure 3:**
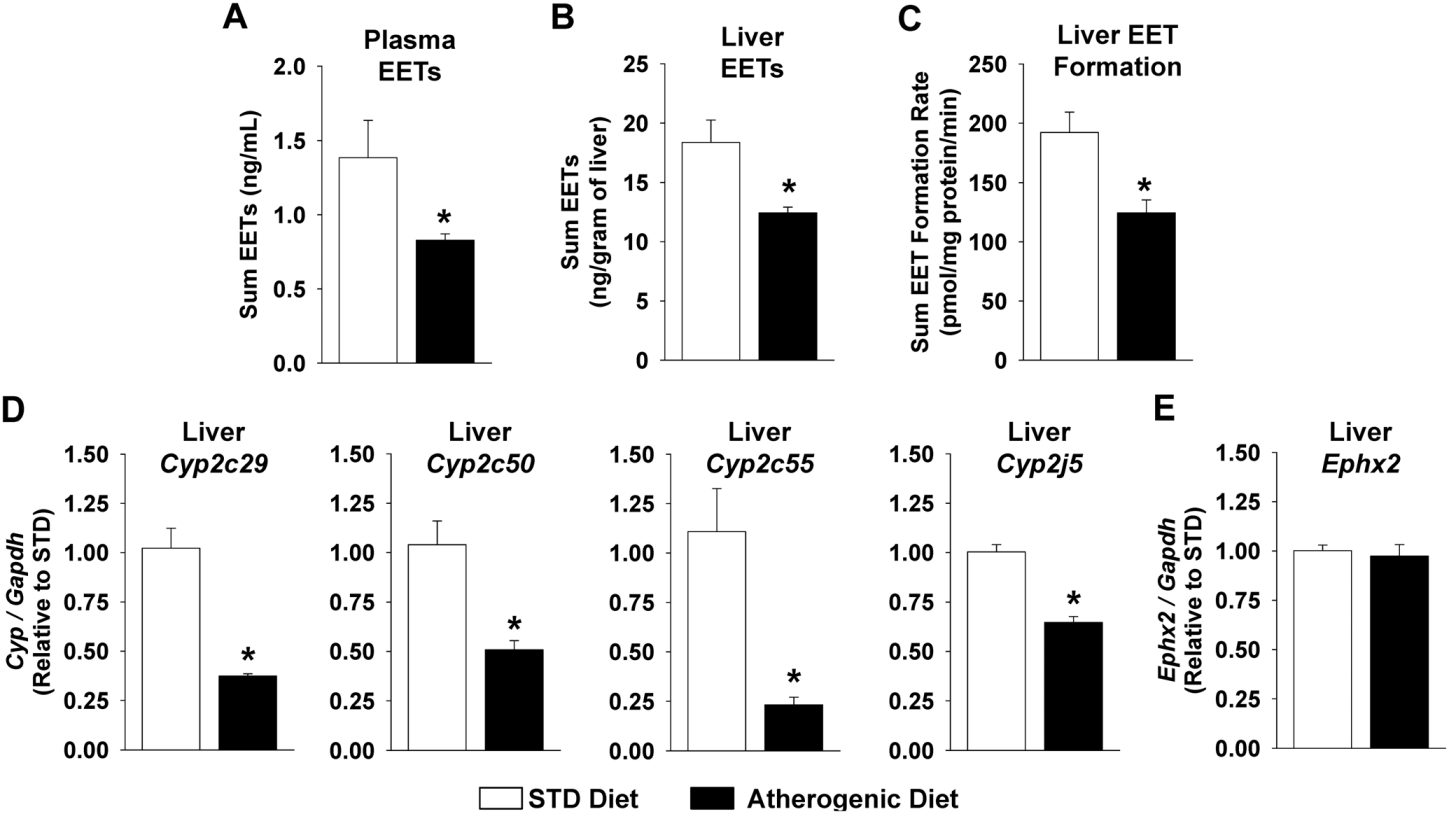
Atherogenic diet administration suppresses the CYP epoxygenase pathway. (A) Plasma sum EET levels and (B) liver sum EET levels were significantly lower in mice administered the atherogenic diet compared to mice administered the STD diet (n = 4–6 per group). (C) The EET formation rate in the presence of saturating arachidonic acid concentrations was also suppressed in liver microsomes isolated from mice administered the atherogenic diet compared to mice administered the STD chow diet (n = 8 per group). (D) Liver *Cyp2c29*, *Cyp2c50*, *Cyp2c55*, *and Cyp2j5* mRNA levels were significantly suppressed but (E) *Ephx2* mRNA levels were not significantly different (STD diet: n = 6, atherogenic diet n = 5). *P<0.05 vs. STD diet group.

In order to specifically investigate the functional effects of fatty liver disease-associated inflammation on CYP-mediated eicosanoid metabolism, we subsequently quantified plasma and hepatic eicosanoid levels and eicosanoid formation rates in liver microsomes. Atherogenic diet administration significantly decreased plasma and liver EET levels *in vivo* (Figure 3A and 3B), which were highly correlated (r_s_ = 0.817, p = 0.007). In addition, hepatic sum EET formation *ex vivo* was significantly suppressed in microsomes isolated from mice fed the atherogenic diet (Figure 3C). A similar suppression of the individual 8,9-, 11,12-, and 14,15-EET regioisomers *in vivo* and *ex vivo* was observed in atherogenic diet fed mice (Figure S1). Consistent with suppression of hepatic CYP epoxygenase metabolic activity, liver *Cyp2c29*, *Cyp2c50*, *Cyp2c55*, and *Cyp2j5* mRNA levels were all significantly suppressed in mice administered the atherogenic diet (Figure 3D). In contrast, no significant differences in hepatic *Ephx2* mRNA levels (Figure 3E) were observed. Furthermore, disparate effects on the expression of Cyp4a and Cyp4f isoforms in liver were observed, with certain isoforms either suppressed, unchanged or induced in response to the atherogenic diet (Figure S2). Consequently, atherogenic diet administration did not significantly impact CYP ω-hydroxylase pathway metabolic function, such that plasma 20-HETE levels (1.5±0.3 vs. 1.3±0.2 ng/ml, p = 0.825), liver 20-HETE levels (4.1±0.3 vs. 3.4±0.4 ng/g, p = 0.218) and liver microsome 20-HETE formation rates (375±27 vs. 338±25 pmol/mg protein/minute, p = 0.689) were similar in mice fed the standard and atherogenic diets, respectively. Collectively, these data suggest that induction of fatty liver disease-associated inflammation dysregulates the arachidonic acid metabolism pathway by preferentially suppressing hepatic CYP epoxygenase metabolic function and hepatic and systemic EET levels.

Furthermore, consistent with activation of the innate immune-mediated inflammatory response and suppression of the CYP epoxygenase pathway, a significant inverse correlation between sum EET concentrations in liver tissue and hepatic *Tlr4* (r_s_ = −0.646, p = 0.032), *Nfκb1* (r_s_ = −0.746, p = 0.009), and *Tnfa* (r_s_ = −0.736, p = 0.010) mRNA levels was observed. Similar inverse correlations were observed with hepatic *Cyp2c29*, *Cyp2c50*, *Cyp2c55*, and *Cyp2j5* mRNA levels (Table S3).

### Hydrodynamic Delivery of *Ephx2* to *Ephx2* ^−/−^ Mice

**Figure 4:**
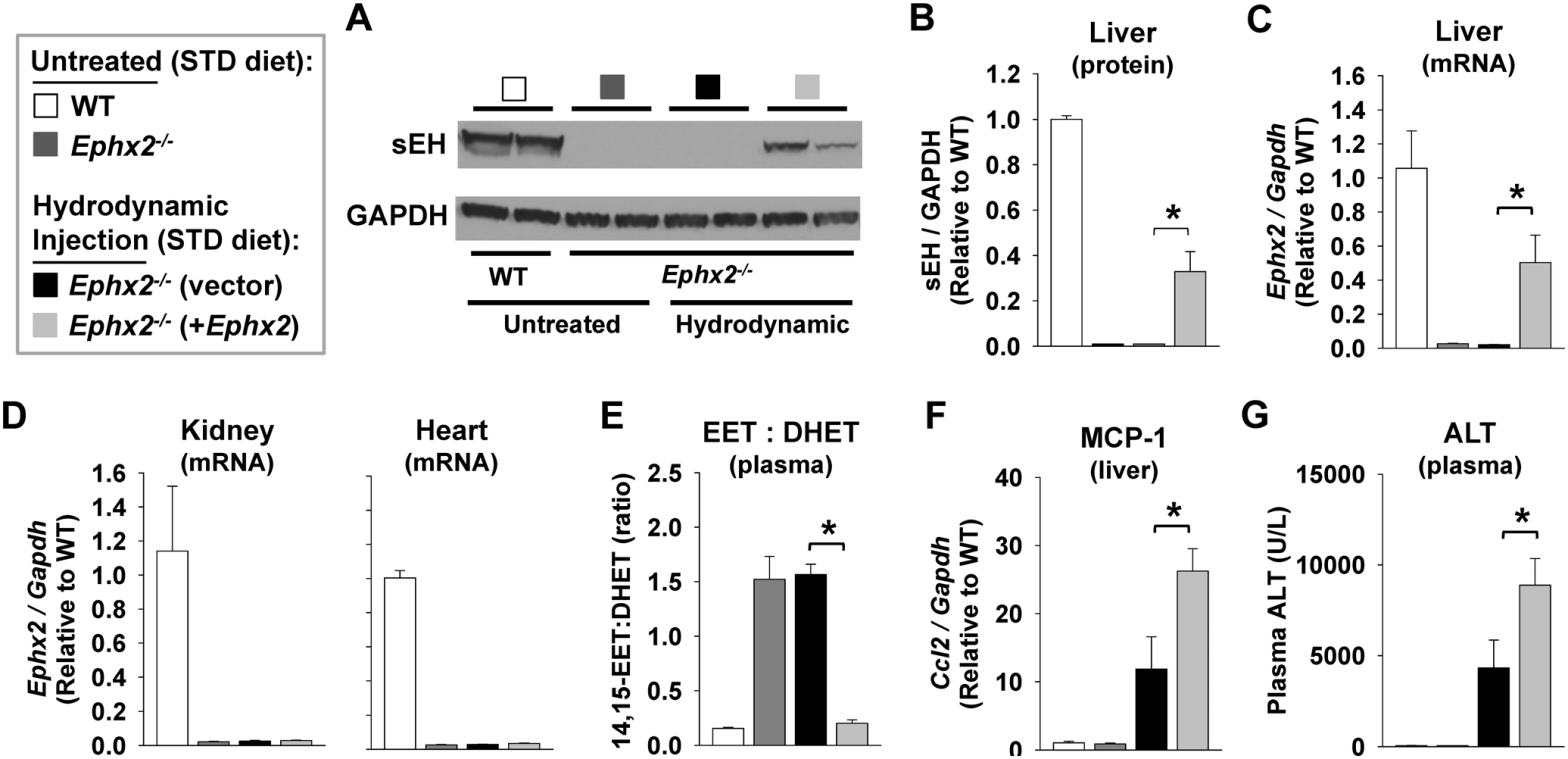
Hydrodynamic delivery of *Ephx2* to *Ephx2^−/−^* mice. *Ephx2* ***^−/−^*** mice were administered plasmid DNA (empty vector [control] or vector containing murine *Ephx2*) by hydrodynamic injection (9% of body weight over 5****seconds). All mice were fed with a standard chow diet, and liver tissue and plasma were harvested 18 hours following injection. A parallel group of untreated WT and *Ephx2* ***^−/−^*** mice were included for comparison (n = 4 per group). (A) A representative immunoblot in liver lysates and (B) corresponding densitometry analysis demonstrate partial restoration of hepatic sEH protein expression in *Ephx2* ***^−/−^*** mice. (C) Quantitative RT-PCR demonstrates partial restoration of *Ephx2* mRNA levels in liver. (D) In contrast, no detectable increase in *Ephx2* mRNA levels was observed in either kidney or heart tissue. (E) Hydrodynamic delivery of *Ephx2* significantly lowered the plasma 14,15-EET:DHET ratio (biomarker of sEH metabolic function [lower ratio indicative of higher function]) to a level equivalent with untreated WT mice. Hydrodynamic injection of empty vector markedly increased (F) hepatic *Ccl2* mRNA levels and (G) plasma ALT levels compared to untreated controls, and induction of these biomarkers of hepatic inflammation and injury were significantly increased in the presence of the *Ephx2* transgene. *P<0.05 vs. *Ephx2* ***^−/−^*** (vector) control.

Due to the strong correlation between hepatic and circulating EET levels, we also sought to characterize the functional contribution of hepatic sEH to circulating EET levels *in vivo*. Delivery of plasmid DNA containing murine *Ephx2* using the hydrodynamic injection-based transfection method elicited partial restoration of hepatic *Ephx2* mRNA (Figure 4C) and hepatic sEH protein levels (Figure 4A and 4B) to *Ephx2^−/−^* mice. In contrast, no detectable increase in renal or myocardial *Ephx2* mRNA levels was observed (Figure 4D). Consistent with disruption of sEH-mediated EET hydrolysis, the plasma 14,15-EET:DHET ratio was significantly higher in *Ephx2^−/−^* (1.52±0.21) compared to WT mice (0.16±0.01) under basal, standard diet fed conditions (p<0.001). Hydrodynamic delivery of *Ephx2* to *Ephx2^−/−^* mice, however, significantly lowered the plasma 14,15-EET:DHET ratio to a level comparable with untreated WT mice (Figure 4E), demonstrating that hepatic sEH is a major contributor to circulating EET levels.

Furthermore, due to the rapid increase in intravascular volume and pressure, the hydrodynamic injection procedure also elicits acute but transient hepatic inflammation and injury that resolves within 24–48 hours [21]. No significant differences in either hepatic MCP-1 expression (p = NS) or plasma ALT levels (p = NS) were observed between *Ephx2^−/−^* and WT mice under basal, standard diet fed conditions (Figure 4F and 4G). However, the acute induction of hepatic MCP-1 (Figure 4F) and plasma ALT levels (Figure 4G) elicited by the hydrodynamic injection was exacerbated in the presence of *Ephx2* transgene expression, suggesting a functional contribution of sEH to the induction of hepatic inflammation and injury.

### Atherogenic Diet Evoked Hepatic Inflammation and Injury in *Ephx2* ^−/−^ Mice

**Figure 5:**
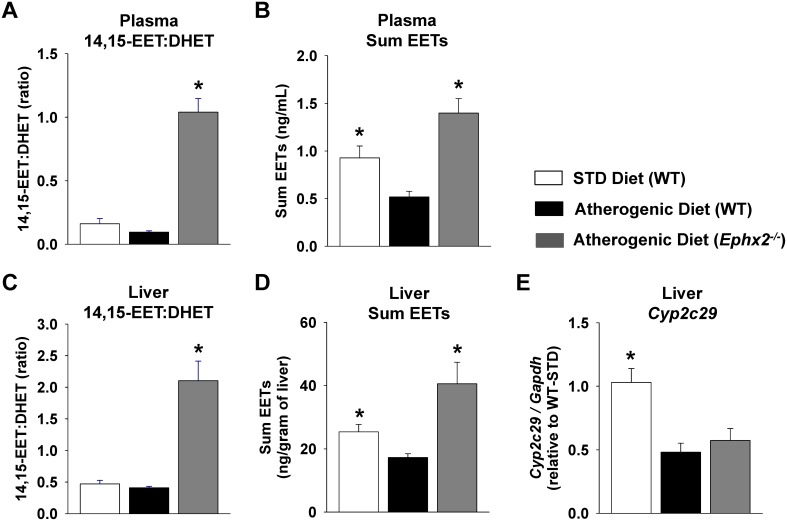
Genetic disruption of sEH-mediated EET hydrolysis increases circulating and hepatic EET levels. Plasma (n = 10–19 per group) and liver (n = 3–5 per group) eicosanoids were quantified in wild-type (WT) mice fed the STD diet and WT and *Ephx2* ***^−/−^*** mice fed the atherogenic diet. (A) Plasma and (C) hepatic 14,15-EET:DHET ratios (biomarker of sEH metabolic function [lower ratio indicative of higher function]) and (B) plasma and (D) hepatic sum EET levels were significantly higher in atherogenic diet fed *Ephx2* ***^−/−^*** compared to WT mice. (E) Liver *Cyp2c29* expression (n = 10–19 per group) was significantly suppressed in both WT and *Ephx2* ***^−/−^*** mice fed the atherogenic diet. *P<0.05 vs. WT atherogenic diet group.

In order to evaluate the functional contribution of the CYP epoxygenase pathway to the regulation of fatty liver disease-associated hepatic inflammation and injury, we administered the atherogenic diet to *Ephx2^−/−^* mice. Consistent with global disruption of sEH-mediated EET hydrolysis, the 14,15-EET:DHET ratio and the sum levels of EETs were significantly higher in atherogenic diet fed *Ephx2^−/−^* compared to atherogenic diet fed WT mice in both plasma and liver (Figure 5A–D). Similar results were observed across the individual EET regioisomers and the 11,12-EET:DHET and 8,9-EET:DHET ratios in both plasma and liver (Table S4). However, *Ephx2* disruption did not influence the atherogenic diet-evoked suppression of CYP epoxygenase expression (Figure 5E, Table S4).

In order to characterize the effects of *Ephx2* disruption on systemic and hepatic cholesterol levels, we first quantified plasma and liver total cholesterol levels, which were significantly increased in response to atherogenic diet administration (p<0.001 vs. standard diet). However, plasma (218±5 vs. 223±6 mg/dL, p = 0.533) and liver (4.0±0.4 vs. 4.2±0.1, p = 0.350) total cholesterol levels were similar in atherogenic diet fed *Ephx2^−/−^* compared to WT mice.

**Figure 6:**
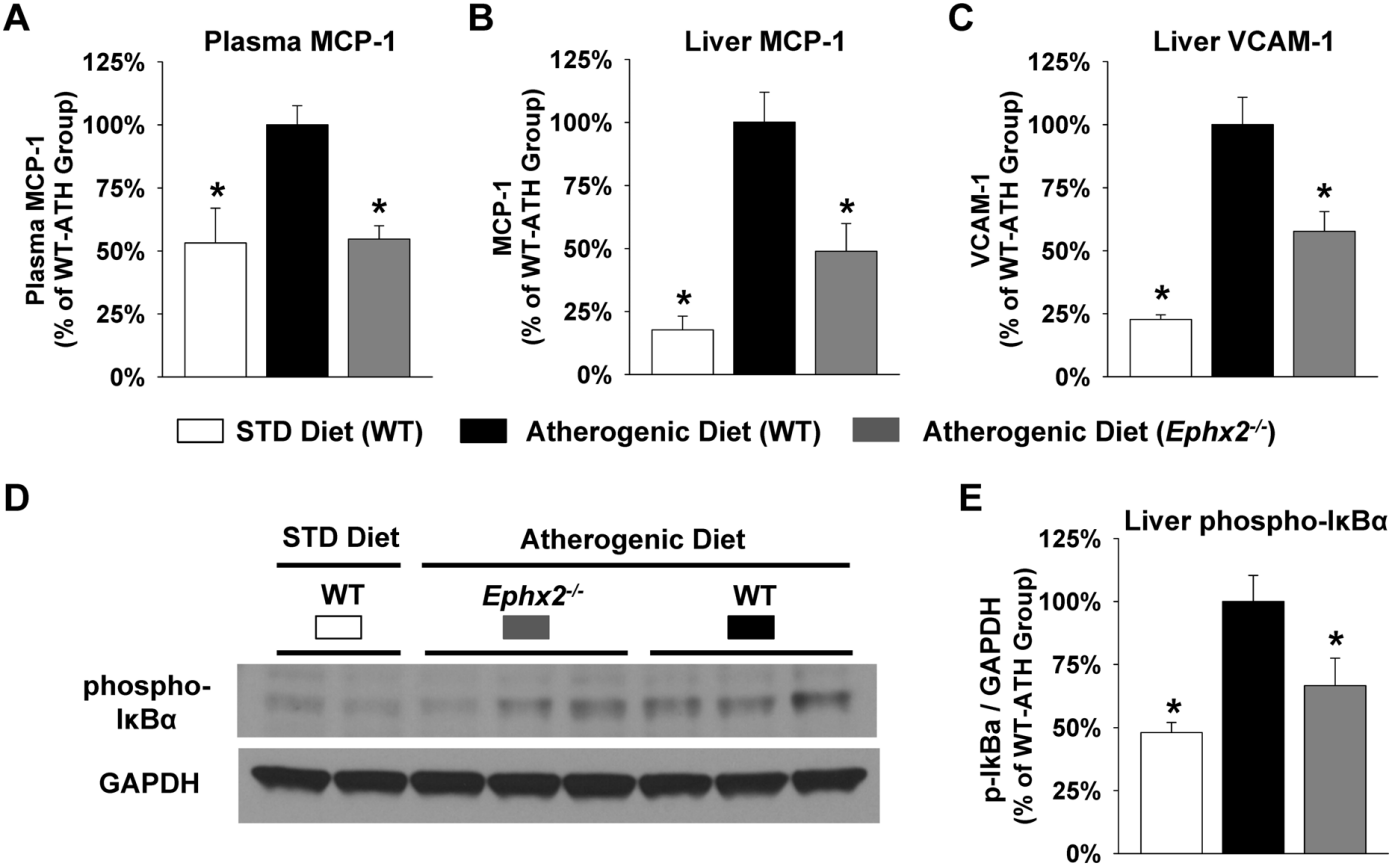
Genetic disruption of sEH attenuates atherogenic diet-induced hepatic expression of inflammatory biomarkers. The atherogenic diet evoked induction of (A) plasma MCP-1, (B) liver MCP-1, and (C) liver VCAM-1 protein levels was significantly attenuated in *Ephx2* ***^−/−^*** mice (WT STD diet: n = 15–16, WT atherogenic diet: n = 37–40, *Ephx2* ***^−/−^*** atherogenic diet: n = 22–24). (D) A representative immunoblot characterizing protein expression of phosphorylated IκB-α and GAPDH in liver lysates is provided. (E) Densitometry analysis demonstrated that the atherogenic diet-induced increase in phosphorylated IκB-α expression, normalized to GAPDH, was attenuated in *Ephx2* ***^−/−^*** mice (n = 4–6 per group). Data are expressed relative to the WT atherogenic diet group. *P<0.05 vs. WT atherogenic diet group.

**Figure 7:**
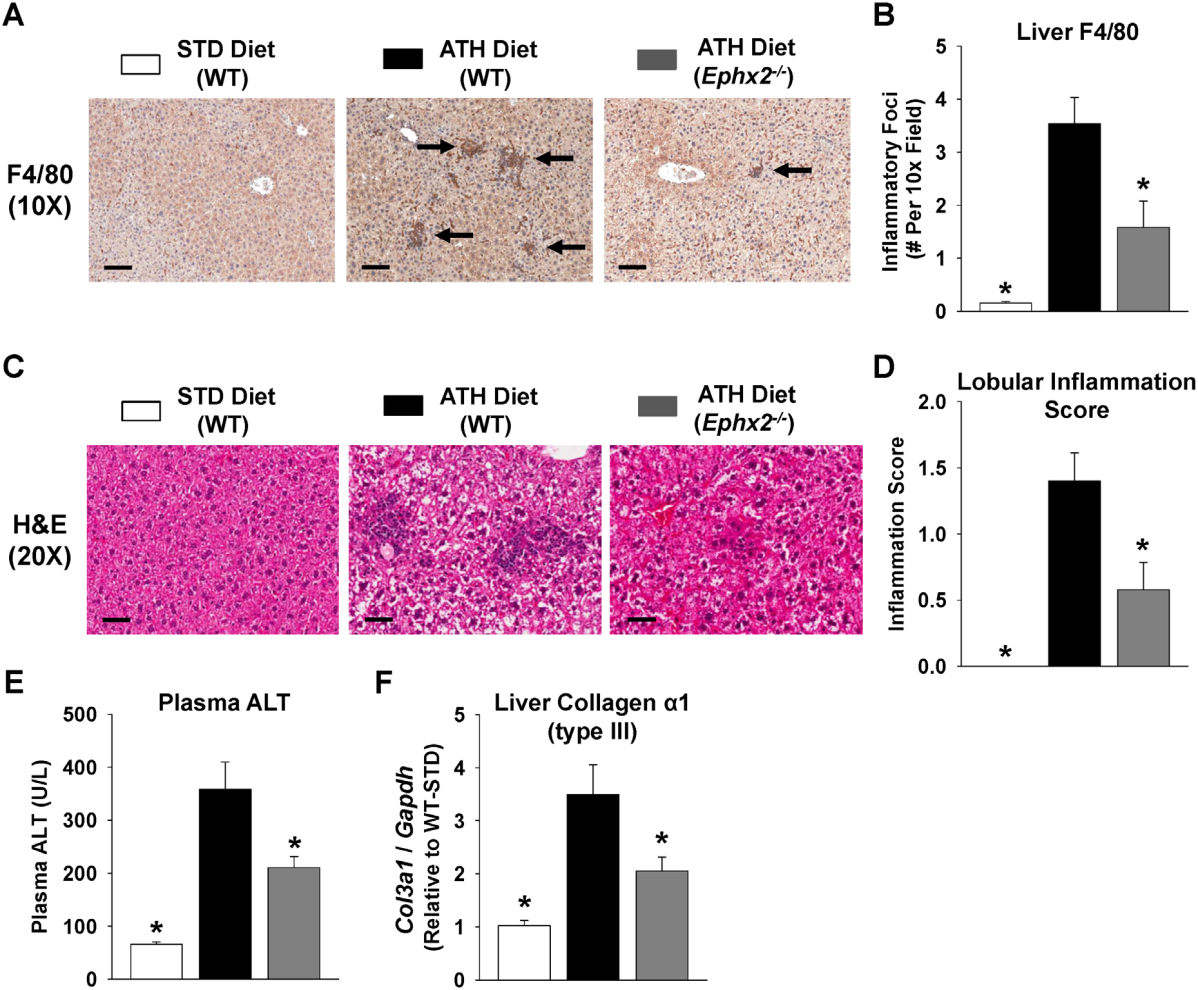
Genetic disruption of sEH attenuates hepatic inflammation and injury. Representative (A) F4/80 (10x, scale bar = 200 **µ**m) and (C) H&E stained (20x, scale bar = 100 **µ**m) images are provided. The atherogenic (ATH) diet induced increase in the (B) number of inflammatory foci per 10x field and (D) lobular inflammation score expressed as a continuous variable was significantly attenuated in *Ephx2* ***^−/−^*** mice (WT STD diet: n = 11–12, WT ATH diet: n = 30–31, *Ephx2* ***^−/−^*** ATH diet: n = 18–19). (E) Induction of plasma ALT levels (WT STD diet: n = 10, WT ATH diet: n = 21, *Ephx2* ***^−/−^*** ATH diet: n = 14) and hepatic collagen (type III, *Col3a1*) expression (WT STD diet: n = 5, WT ATH diet: n = 15, *Ephx2* ***^−/−^*** ATH diet: n = 10) by the ATH diet were significantly attenuated in *Ephx2* ***^−/−^*** mice. *P<0.05 vs. WT ATH diet group.

We next evaluated expression of key inflammatory mediators in WT and *Ephx2^−/−^* mice. Compared to the standard chow diet, administration of the atherogenic diet significantly increased plasma MCP-1, hepatic MCP-1, and hepatic VCAM-1 protein levels (Figure 6A–C). The induction of chemokine and cellular adhesion molecule expression, however, was significantly attenuated in the *Ephx2^−/−^* mice. The atherogenic diet-evoked induction of NF-κB activation in liver tissue was also significantly attenuated in *Ephx2^−/−^* mice (Figure 6D and 6E). Furthermore, WT mice fed the atherogenic diet exhibited significant infiltration of macrophages into liver tissue compared to standard diet fed mice (Figure 7A and 7B); however, macrophage infiltration was significantly attenuated in the *Ephx2^−/−^* mice.

We further assessed the pathological severity of NAFLD/NASH through histological evaluation of lobular inflammation [31]. Lobular inflammation was absent in all mice fed the standard diet and present in the vast majority of WT mice administered the atherogenic diet (Table 1). The atherogenic diet group had a significantly higher inflammation score compared to the standard diet group, and *Ephx2^−/−^* mice had significantly lower inflammation scores compared to WT mice fed the atherogenic diet (Figure 7C and 7D). An ordinal logistic regression analysis confirmed that lobular inflammation scores were significantly lower in *Ephx2^−/−^* compared to WT mice administered the atherogenic diet (Table 1). Furthermore, induction of plasma ALT levels, a biomarker of hepatic injury and necrosis, and hepatic collagen (type III) expression, an early biomarker of collagen deposition, were significantly attenuated in *Ephx2^−/−^* compared to WT mice (Figure 7E and 7F).

**Table 1:** Hepatic lobular inflammation scores.

Lobular Inflammation Score (0–3)					
Group	0	1	2	3	
Standard Diet					
WT (control)	11 (100%)	0 (0%)	0 (0%)	0 (0%)	
Atherogenic Diet					
WT	9 (30%)	7 (23%)	7 (23%)	7 (23%)	
*Ephx2^−/−^*	12 (63%)	4 (21%)	2 (11%)	1 (2%)	p = 0.034^a^

Data presented as the n (percentage) of mice from each group having an inflammation score of 0, 1, 2, or 3. The percentages may not equal 100% due to rounding.

a P-value for the ordinal logistic regression analysis evaluating the impact of genotype (*Ephx2^−/−^* vs. WT) on lobular inflammation score (coded as an ordinal variable: 0, 1, 2, 3) in mice administered the atherogenic diet.

## Discussion

Fatty liver disease is a rapidly growing public health problem without effective therapies that confers significant morbidity and mortality [1], and sustained activation of the innate immune inflammatory response is a key pathologic driver of its progression [4]. Accumulating evidence has demonstrated that CYP-mediated eicosanoid metabolism is an important regulator of acute and chronic inflammatory responses, and promoting the effects of CYP epoxygenase-derived EETs has been proposed as an anti-inflammatory therapeutic strategy for cardiometabolic and renal diseases [15]. However, despite the integral pathologic role of hepatic inflammation in NAFLD/NASH and the abundance of CYP enzyme expression in the liver, the contribution of CYP-mediated eicosanoid metabolism to the pathogenesis and progression of this emerging public health problem remains largely unexplored. Using the atherogenic diet model of NAFLD/NASH, this study is the first to demonstrate that 1) induction of NAFLD/NASH markedly and preferentially suppresses hepatic EET biosynthesis and circulating EET levels through suppression of hepatic CYP epoxygenase expression; and, 2) targeted disruption of *Ephx2* restores hepatic and systemic EET levels and attenuates NAFLD/NASH-evoked hepatic inflammation and injury. Collectively, these findings suggest that suppression of hepatic EET biosynthesis is a key pathological consequence of NAFLD/NASH, and therapeutic restoration of EET levels is an anti-inflammatory strategy with potential utility for the treatment of fatty liver disease-associated inflammation and injury.

It is well-established that inflammatory stimuli suppress hepatic CYP-mediated xenobiotic metabolism [32]. In addition, we have reported that hepatic EET biosynthesis is suppressed in an LPS model of acute inflammation and a high-fat diet model of insulin resistance [12], [33]. Although accumulating evidence has demonstrated that NAFLD/NASH dysregulates hepatic CYP-mediated xenobiotic metabolism, and these effects are largely CYP isoform-specific [34], the impact on CYP-mediated eicosanoid metabolism has not been evaluated to date. Our expression analysis demonstrated that, relative to global gene expression changes, the arachidonic acid metabolism pathway is significantly dysregulated in liver following atherogenic diet administration. Notably, these changes were largely driven by suppression of CYP expression, including numerous Cyp2c and Cyp2j epoxygenases. Moreover, we confirmed that atherogenic diet administration evoked a marked suppression of hepatic *Cyp2c29*, *Cyp2c50*, *Cyp2c55*, and *Cyp2j5* expression, the most abundant CYP epoxygenases in mouse liver [12]. Furthermore, EET biosynthesis in liver microsomes and both plasma and liver EET levels were significantly suppressed. In contrast, hepatic 20-HETE biosynthesis and hepatic and plasma 20-HETE levels were not altered in mice administered the atherogenic diet, while hepatic expression of key Cyp4a and Cyp4f isoforms were either suppressed (*Cyp4a12*, *Cyp4f13*), unchanged (*Cyp4f15*), or induced (*Cyp4f16*). The disparate effects observed on hepatic CYP ω-hydroxylase expression in response to the atherogenic diet are consistent with previous findings in acute models of inflammation [12], and suggest that the mechanisms underlying regulation of hepatic 20-HETE biosynthesis in the presence of inflammation are complex and require further investigation. Collectively, these data suggest that suppression of hepatic CYP epoxygenase-mediated EET biosynthesis is a key pathological consequence of NAFLD/NASH.

Previous studies have demonstrated that LPS-induced inflammation suppresses hepatic CYP epoxygenase expression *in vivo* [12]. In addition, inflammatory cytokines including IL-1, IL-6 and TNFα suppress CYP expression in hepatocytes, and cytokine-mediated CYP suppression is dependent on NF-κB activation [35]. The direct contribution of specific nuclear receptors to these effects, however, appears to be isoform- and species-specific and model-dependent, which suggests that upstream activation of the innate immune response is the most important factor driving the suppression of hepatic CYP expression. The atherogenic diet model of NAFLD/NASH induces hepatic inflammation via an innate immune system dependent mechanism [7], [8]. Interestingly, we observed a significant inverse correlation between *Tlr4, Nfκb1,* and *Tnfα* expression and both hepatic CYP epoxygenase expression and EET levels, suggesting that atherogenic diet-evoked suppression of hepatic EET biosynthesis is most likely mediated via activation of the innate immune response and down-regulation of CYP epoxygenase expression. However, future studies remain necessary to elucidate the mechanisms underlying these effects.

Despite the abundant expression of CYP epoxygenases and sEH in liver, the contribution of hepatic EET biosynthesis and hydrolysis to circulating EET levels has remained unclear. Interestingly, plasma and liver EET levels were highly correlated in our atherogenic diet experiments. In addition, our *Ephx2* transgene delivery experiment demonstrated that restoration of hepatic sEH expression in *Ephx2^−/−^* mice restored the plasma 14,15-EET:DHET ratio to levels similar to those observed in WT mice. Taken together, these data suggest that EET biosynthesis and hydrolysis in the liver is a major contributor to circulating EET levels. The link between hepatic and plasma EET levels may have important implications for other inflammatory diseases, as well as the utility of circulating EET levels and the 14,15-EET:DHET ratio as a metabolic biomarker in human studies, as plasma EET levels and the plasma 14,15-EET:DHET ratio were inversely correlated with circulating biomarkers of inflammation in humans with stable coronary artery disease [36]. Although lipidomic analyses in humans have demonstrated that NAFLD/NASH is associated with dysregulated fatty acid metabolism, [37] the relationship between the presence and severity of NAFLD/NASH and altered CYP-derived eicosanoid metabolites has not been evaluated in humans to date. Our findings demonstrate the need, and lay the foundation, for future translational research in this area.

It is now well-established that CYP-derived EETs have potent anti-inflammatory effects in preclinical models of NF-κB-mediated vascular inflammation [12], [38], and also exhibit anti-apoptotic and anti-fibrotic properties [15], [39], [40]; therefore, the observed suppression of hepatic EET biosynthesis may propagate the inflammatory response and thus be detrimental in the pathological progression of NAFLD/NASH. Although recent reports indicate that sEH inhibition may attenuate the development of insulin resistance and hepatic steatosis in response to a high-fat diet [41], [42], the functional contribution of the CYP epoxygenase pathway to the regulation of NAFLD/NASH-associated hepatic inflammation and injury had not been evaluated. We hypothesized that decreasing sEH-mediated EET hydrolysis, by targeted disruption of *Ephx2*, would restore hepatic EET levels and attenuate atherogenic diet induced hepatic inflammation and injury. Consistent with our hypothesis and the anti-inflammatory effects of EETs, *Ephx2^−/−^* mice exhibited increased hepatic and circulating EET levels and significantly attenuated NF-κB activation, hepatic chemokine and cellular adhesion molecule expression, macrophage infiltration into liver tissue, hepatic injury, and collagen activation in response to atherogenic diet feeding. Collectively, these findings demonstrate that sEH is an important regulator of NAFLD/NASH-associated hepatic inflammation and injury. In addition, we observed that acute hepatic inflammation and injury evoked by the hydrodynamic injection procedure was exacerbated in the presence of hepatic *Ephx2* transgene expression, further implicating sEH as a key regulator of hepatic inflammation and injury.

Although the atherogenic diet model of NAFLD/NASH increases circulating and hepatic cholesterol levels, increases hepatic triglycerides, activates the innate immune system, and induces hepatic inflammation and injury within four weeks, these effects are not driven by weight gain or systemic insulin resistance. Thus, the atherogenic diet has limitations as a model of human NAFLD/NASH, where the most common underlying etiology is chronic obesity and insulin resistance. However, due to the known insulin sensitizing effects of sEH inhibition [41], [42], use of the atherogenic diet model allowed us to evaluate the fundamental pathological role of hepatic inflammation in NAFLD/NASH without the confounding effects of weight gain, adipose tissue inflammation, and insulin resistance. Moreover, sEH is a bifunctional enzyme with both an epoxide hydrolase and lipid phosphatase domain [43]. Genetic disruption of sEH, which abolishes both domains, has been reported to lower basal plasma cholesterol levels [44]; however, in the present study genetic disruption of sEH did not have an effect on the atherogenic diet induced increase in plasma or hepatic cholesterol levels. Therefore, it is unlikely that changes in cholesterol metabolism impacted the anti-inflammatory phenotypes observed in the *Ephx2^−/−^* mice. Follow-up studies evaluating the effects of *Ephx2* disruption and pharmacologic strategies that promote the effects of EETs, including sEH inhibitors and stable EET analogs, in additional models of NAFLD/NASH remain necessary to elucidate the functional contribution of CYP-derived EETs to fatty liver disease.

### Conclusions

In summary, we have demonstrated that hepatic CYP epoxygenase activity, and hepatic and circulating EET levels, is significantly and preferentially suppressed in the atherogenic diet model of NAFLD/NASH. In addition, genetic disruption of sEH increased EET levels and attenuated atherogenic diet induced hepatic inflammation and injury. Collectively, these data suggest that suppression of hepatic CYP-mediated EET biosynthesis is an important pathological consequence of NAFLD/NASH, and indicate that the CYP epoxygenase pathway is an important regulator of the NAFLD/NASH-associated hepatic inflammatory response *in vivo*. Future studies are needed to evaluate the therapeutic utility of strategies that promote the effects of CYP-derived EETs in NAFLD/NASH, and improve our understanding of the mechanisms underlying the contribution of sEH and EETs to the regulation of NAFLD/NASH-associated chronic hepatic inflammation.

## Supporting Information

Figure S1 **Effects of atherogenic diet on the CYP epoxygenase pathway by EET regioisomer.**
(PDF)

Figure S2 **Effects of atherogenic diet on the CYP ω-hydroxylase pathway.**
(PDF)

Table S1 **Probes significantly changed in liver following atherogenic diet feeding in wild-type mice (rank ordered by P-value).**
(XLSX)

Table S2 **Functional classification of genes expressed in liver that were significantly dysregulated in response to atherogenic diet feeding in wild-type mice.**
(PDF)

Table S3 **Correlation between hepatic mRNA levels of CYP epoxygenases and mediators of the innate immune inflammatory response in mice.**
(PDF)

Table S4 **Effects of atherogenic diet and** ***Ephx2*** **disruption on circulating and hepatic EET levels by regioisomer.**
(PDF)
