# Supplementary material for: The Cytochrome P450 Epoxygenase Pathway Regulates the Hepatic Inflammatory Response in Fatty Liver Disease
Source: PLoS One. 2014 Oct 13;9(10):e110162. doi: 10.1371/journal.pone.0110162 (PMC4195706; doi:10.1371/journal.pone.0110162)
Supplement: Figure S1 — Effects of atherogenic diet on the CYP epoxygenase pathway by EET regioisomer. (PDF) [file pone.0110162.s001.pdf]

Figure S1

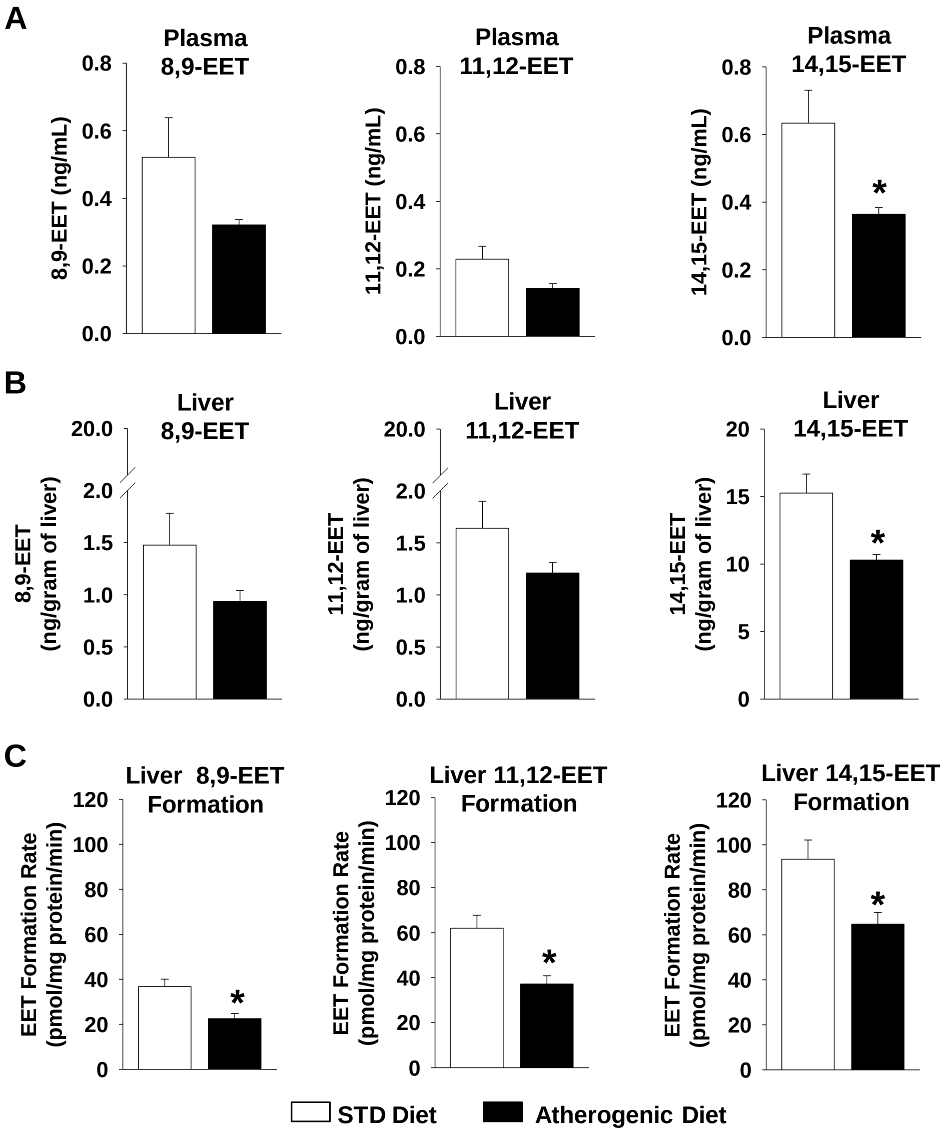

Figure S1. Effects of atherogenic diet on the CYP epoxygenase pathway by EET regioisomer. Consistent with the sum EET data presented in Figure 3, (A) plasma and (B) liver concentrations of the 8,9-, 11,12-, and 14,15-EET regioisomers *in vivo* were suppressed in response to the atherogenic diet (n=4-6 per group). (C) The 8,9-, 11,12, and 14,15-EET regioisomer formation rates in the presence of saturating arachidonic acid concentrations were each significantly suppressed in liver microsomes isolated from mice administered the atherogenic diet compared to mice administered the STD chow diet (n=8 per group). *P<0.05 vs. STD diet group.
