# Supplementary material for: The Cytochrome P450 Epoxygenase Pathway Regulates the Hepatic Inflammatory Response in Fatty Liver Disease
Source: PLoS One. 2014 Oct 13;9(10):e110162. doi: 10.1371/journal.pone.0110162 (PMC4195706; doi:10.1371/journal.pone.0110162)
Supplement: Figure S2 — Effects of atherogenic diet on the CYP ω-hydroxylase pathway. (PDF) [file pone.0110162.s002.pdf]

Figure S2

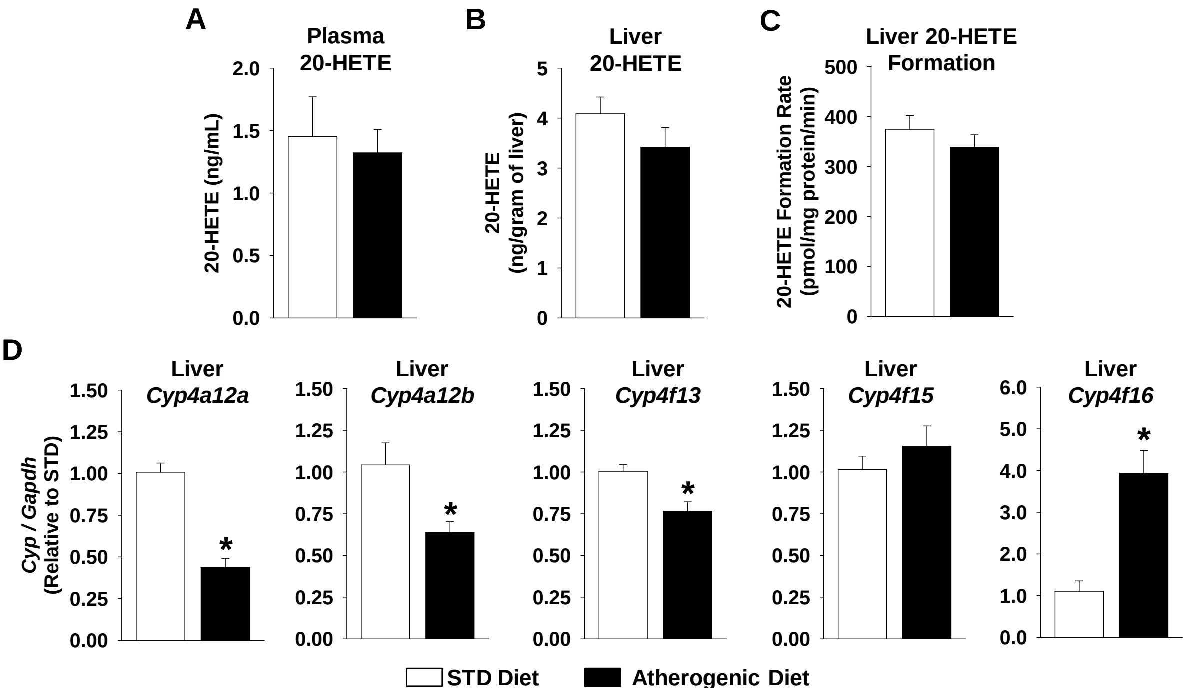

Figure S2. Effects of atherogenic diet on the CYP ω-hydroxylase pathway. (A) Plasma and (B) liver 20-HETE levels (n=4-6 per group) were not significantly altered in response to the atherogenic diet. (C) The 20-HETE formation rate in the presence of saturating arachidonic acid concentrations was not significantly altered in liver microsomes isolated from mice administered the atherogenic diet compared to mice administered the STD chow diet (n=8 per group). (D) Liver *Cyp4a12a*, *Cyp4a12b*, and *Cyp4f13* mRNA levels were significantly suppressed, whereas *Cyp4f15* mRNA levels were not altered and *Cyp4f16* mRNA levels were significantly induced in WT mice fed the atherogenic diet (n=5-6 per group). *P<0.05 vs. STD diet group.
