# Supplementary material for: The Cytochrome P450 Epoxygenase Pathway Regulates the Hepatic Inflammatory Response in Fatty Liver Disease
Source: PLoS One. 2014 Oct 13;9(10):e110162. doi: 10.1371/journal.pone.0110162 (PMC4195706; doi:10.1371/journal.pone.0110162)
Supplement: Table S2 — Functional classification of genes expressed in liver that were significantly dysregulated in response to atherogenic diet feeding in wild-type mice. (PDF) [file pone.0110162.s004.pdf]

Table S2. Functional classification of genes expressed in liver that were significantly dysregulated in response to atherogenic diet feeding in wild-type mice.

**Cluster**	**Fold-Enrichment**	**P-Value**
Pyridoxal phosphate	3.56	<0.0001
Coenzyme dependent metabolism	4.97	<0.0001
Purine nucleotide binding	1.30	<0.0001
Cytochromes P450	2.70	0.0001
Phagocytosis / endocytosis	3.81	0.0003
Purine nucleoside binding	1.29	0.0004
Arginine dependent process	7.67	0.0018
Histidine box	5.89	0.0071
Oligoadenylate synthetase	5.78	0.0087

"Cluster" provides a description of each functional annotation cluster that exhibited significant pathway enrichment ($p<0.01$, Enrichment Score >2) in response to atherogenic diet feeding. The functional annotation clusters are presented in rank order by p-value.

"Fold enrichment" indicates the number of genes within each functional annotation cluster relative to the number of genes that would be expected by chance.

The p-value for each functional annotation cluster is provided.
