# Supplementary material for: The Cytochrome P450 Epoxygenase Pathway Regulates the Hepatic Inflammatory Response in Fatty Liver Disease
Source: PLoS One. 2014 Oct 13;9(10):e110162. doi: 10.1371/journal.pone.0110162 (PMC4195706; doi:10.1371/journal.pone.0110162)
Supplement: Table S3 — Correlation between hepatic mRNA levels of CYP epoxygenases and mediators of the innate immune inflammatory response in mice. (PDF) [file pone.0110162.s005.pdf]

Table S3. Correlation between hepatic mRNA levels of CYP epoxygenases and mediators of the innate immune inflammatory response in mice.

	Cyp2c29	*Cyp2c50*	*Cyp2c55*	*Cyp2j5*
Tlr4	-0.63 (0.035)	-0.62 (0.043)	-0.69 (0.019)	-0.86 (0.001)
Nfkb1	-0.75 (0.007)	-0.74 (0.010)	-0.65 (0.029)	-0.59 (0.056)
Tnfa	-0.70 (0.017)	-0.63 (0.039)	-0.75 (0.007)	-0.69 (0.019)

Data presented as Spearman correlation coefficient (p-value).
