# Supplementary material for: The Cytochrome P450 Epoxygenase Pathway Regulates the Hepatic Inflammatory Response in Fatty Liver Disease
Source: PLoS One. 2014 Oct 13;9(10):e110162. doi: 10.1371/journal.pone.0110162 (PMC4195706; doi:10.1371/journal.pone.0110162)
Supplement: Table S4 — Effects of atherogenic diet and Ephx2 disruption on circulating and hepatic EET levels by regioisomer. (PDF) [file pone.0110162.s006.pdf]

Table S4. Effects of atherogenic diet and *Ephx2* disruption on circulating and hepatic EET levels by regioisomer.

Eicosanoid	Standard Diet *Wild-Type*	Atherogenic Diet *Wild-Type*	Atherogenic Diet *Ephx2$^{-/-}$*
**Plasma**			
EET:DHET ratios			
8,9-EET:DHET	0.19 ± 0.07	0.06 ± 0.01	0.46 ± 0.08 *
11,12-EET:DHET	0.10 ± 0.05	0.04 ± 0.01	0.24 ± 0.08 *
14,15-EET:DHET	*See Figure 5A*		
EET regioisomers (ng/mL)			
8,9-EET	0.26 ± 0.05 *	0.12 ± 0.02	0.29 ± 0.04 *
11,12-EET	0.18 ± 0.06	0.09 ± 0.01	0.17 ± 0.04 *
14,15-EET	0.49 ± 0.04	0.31 ± 0.04	0.93 ± 0.12 *
Sum EETs	*See Figure 5B*		
**Liver**			
EET:DHET ratios			
8,9-EET:DHET	0.32 ± 0.05	0.15 ± 0.02	0.55 ± 0.13 *
11,12-EET:DHET	0.10 ± 0.01	0.08 ± 0.04	0.10 ± 0.02
14,15-EET:DHET	*See Figure 5C*		
EET regioisomers (ng/g tissue)			
8,9-EET	2.47 ± 0.58 *	1.07 ± 0.09	3.24 ± 0.30 *
11,12-EET	2.56 ± 0.46	2.40 ± 1.00	3.38 ± 0.85
14,15-EET	20.3 ± 1.66 *	14.0 ± 0.90	34.0 ± 5.93 *
Sum EETs	*See Figure 5D*		
CYP epoxygenase expression (relative)			
Cyp2j5	1.00 ± 0.08 *	0.64 ± 0.08	0.72 ± 0.10
Cyp2c50	1.00 ± 0.08 *	0.44 ± 0.07	0.53 ± 0.10
Cyp2c29	*See Figure 5E*		

Plasma (n=10-19 per group) and liver (n=3-5 per group) eicosanoids and liver CYP epoxygenase expression (n=8 per group) were quantified in wild-type (WT) mice fed the standard diet and WT and *Ephx2$^{-/-}$* mice fed the atherogenic diet.

Data presented as mean ± SEM.

*P<0.05 versus WT-atherogenic diet group
